# Portuguese ABE’s BPSD score (ABS): exploring agreement between ABS items and neuropsychiatric inventory domains

**DOI:** 10.1192/j.eurpsy.2021.1141

**Published:** 2021-08-13

**Authors:** A.R. Ferreira, C.C. Dias, M.R. Simões, K. Abe, L. Fernandes

**Affiliations:** 1 Cintesis – Center For Health Technology And Services Research, Faculty of Medicine, University of Porto, Porto, Portugal; 2 Department Of Community Medicine, Information And Health Decision Sciences, Faculty of Medicine, University of Porto, Porto, Portugal; 3 Cineicc, Psyassessmentlab, Faculty of Psychology and Educational Sciences, University of Coimbra, Coimbra, Portugal; 4 Department Of Neurology, Graduate School of Medicine, Dentistry and Pharmaceutical Science, Okayama University, Okayama, Japan; 5 Department Of Clinical Neurosciences And Mental Health Department, Faculty of Medicine, University of Porto, Porto, Portugal; 6 Psychiatry Service, Centro Hospitalar Universitário de São João, Porto, Portugal

**Keywords:** Neuropsychiatric symptoms, dementia, Psychiatric Status Rating Scales, Validation study

## Abstract

**Introduction:**

Neuropsychiatric symptoms (NPS) are common, disabling and burdensome core-features of dementia, with important diagnostic and prognostic value. However, their measurement remains challenging. The Neuropsychiatric Inventory (NPI) is the most widely used NPS measure. Nevertheless, it is also time-consuming and impractical in most clinical settings. Therefore, the Abe’s BPSD score (ABS) has been proposed as a brief score to facilitate the NPS assessment.

**Objectives:**

To explore the concurrent validity of the Portuguese ABS by comparing the 10 ABS items with the relevant NPI-12 domains.

**Methods:**

A cross-sectional study was conducted with outpatients attending a gerontopsychiatric consultation. Patients were included if they were ≥65 years and had a reliable caregiver. NPS frequency rates (number of patients with a symptom) were estimated with ABS and NPI-12, and an agreement analysis was undertaken by calculating kappa-coefficients (k) and the respective 95% confidence interval [95%CI] between ABS items and relevant NPI-12 domains.

**Results:**

Overall, 107 patients were included. Kappa-values ranged from 0.277 to 1.000. Higher agreement was recorded for the ABS items eating/toilet problems (k=1.000), day–night reversal (k=0.976[0.925-1.000]) and depressive/gloomy mood (k=0.957[0.899-1.000]), with the NPI-12 appetite/eating abnormalities, night-time behavioural disturbances and dysphoria domains, respectively. The ABS item violent force recorded the lowest agreement (k=0.277[0.104-0.45]) with the NPI-12 agitation/aggression domain.

**Conclusions:**

This exploratory analysis demonstrates good levels of agreement between most ABS items and relevant NPI-12 domains. Data add to the evidence that both measures capture a comparable broad spectrum of psychopathology, supporting the ABS use in clinical routine. Support: FCT(PD/BD/114555/2016), and National Funds through FCT-within CINTESIS, R&D Unit (ref.UIDB/4255/2020).

